# Medical Items

**Published:** 1912-07

**Authors:** 


### MEDICAL ITEMS

### A SUGGESTION IN TETANUS.

The physician who has ever faced the horrors of tetanus and has seen his ministration go for naught, will not hesitate to add to this disease's classic treatment, any agency holding out even the faintest ray of hope. Quite a number of physicians have employed PASADYNE, (Daniel's Concentrated Tincture of Passiflora Incarnata), in tetanus and some have reported favorably on it. It is advised, therefore, that PASADYNE be employed as an adjunct treatment in this disease. It possesses marked calmative powers, and may mitigate the distressing convulsive seizures of this dreadful infection. A sample bottle will be furnshed if application be made to the Laboratory of John B. Daniel, Atlanta, Ga.

---

### RECONSTRUCTION FOLLOWING TYPHOID FEVER.

In some instances, the convalescence of typhoid fever presents a debility closely akin to a tuberculous predisposition, which indicates the need for more potent reconstructive than the stomachics and tonics usually employed for this purpose. This need is well met by Cord. Ext. Morrhuae Comp. (Hagee). Usually in these cases the blood stream is thin, the processes of metabolism are interfered with and the vital powers remain far below par. The tissues are easily susceptible to graver infections, such as tuberculosis. Cord. Ext. Ol. Morrhuae Comp. (Hagee) will prove its worth as an up-builder in this class of cases, charging the blood current with nutritious elements and finally overcoming the debilitated state. Its palatability gives it added utility, a feature worthy of consderation in choosing remedial agents of this character.

rofessor Davenport in his admirable work on Diseases of Woman, Dr. L. G. Boyd, Tunnelton, Ind., reviewing, adds:

How can we prevent dysmennorrhea? It can be done by keeping the patient under morphine but this is a barbarous solution of an important problem. It, in fact, does not solve it. Morphine is inadmissable and improper in these cases. It produces derangement of the secretions and tends to establish a drug habit that will make life a burden. I have long employed a remedy that not only relieves the pain, but produces no habit and is not dangerous. I refer to Dioviburnia. It is a most valuable uterine tonic, antispasmodic and anodyne of exceptional worth. I rely upon this remedy to prevent dysmenorrhea, which as Professor Davenport truly says, is seen in almost all, if not all women. I have my patients who suffer with dysmenorrhea to take Dioviburnia, beginning two days before menstruation is due and persist in it until the period ha spassed. I give it in doses of one to two teaspoonfuls every three hours throughout this time. When this diretcion is followed I have found that my patients go through the period without pain. The adoption of this treatment I may say also, has brought me many grateful compliments.

Where the patient is very nervous, having the tendency to hysteria, neuroses or uterine congestion, I administer Neurosine one part, n combination wth two parts of Doviburnia, which always gives relief.

---

Dr. Katherine L. Storm, who a few years ago patented the Storm Binder, recently obtained patents in England and Canada on this supporter, also another patent in the United States for improvements that have been made to meet the extended requirements for a high belt for floating kidney-ptosis, etc., with a minimum of pressure, heat and weight across the back of the patient.

